# Dietary polyphenols drive dose-dependent behavioral and molecular alterations to repeated morphine

**DOI:** 10.1038/s41598-023-39334-9

**Published:** 2023-07-27

**Authors:** Aya Osman, Rebecca S. Hofford, Katherine R. Meckel, Yesha A. Dave, Sharon M. Zeldin, Ava L. Shipman, Kelsey E. Lucerne, Kyle J. Trageser, Tatsunori Oguchi, Drew D. Kiraly

**Affiliations:** 1 Department of Psychiatry, Icahn School of Medicine at Mount Sinai, New York, NY USA; 2 The Seaver Center for Autism Research and Treatment, Icahn School of Medicine at Mount Sinai, New York, NY USA; 3 Friedman Brain Institute, Icahn School of Medicine at Mount Sinai, New York, NY USA; 4 Department of Physiology, Pharmacology and Psychiatry, Wake Forest School of Medicine, 115 S. Chestnut Street, Winston-Salem, NC 27104 USA; 5 Nash Family Department of Neuroscience, Icahn School of Medicine at Mount Sinai, New York, NY USA; 6 Department of Neurology, Icahn School of Medicine at Mount Sinai, New York, NY USA; 7 Geriatric Research, Education and Clinical Center, James J. Peters Veterans Affairs Medical Center, Bronx, NY USA; 8 Department of Psychiatry, Atrium Health Wake Forest Baptist, Winston-Salem, NC USA

**Keywords:** Molecular neuroscience, Neuroscience, Diseases of the nervous system, Addiction

## Abstract

Opioid Use Disorder (OUD) is associated with tremendous morbidity and mortality. Despite this burden, current pharmacotherapies for OUD are ineffective or intolerable for many patients. As such, interventions aimed at promoting resilience against OUD are of immense clinical interest. Treatment with a Bioactive Dietary Polyphenol Preparation (BDPP) promotes resilience and adaptive neuroplasticity in multiple models of neuropsychiatric disease. Here, we assessed effects of BDPP treatment on behavioral and molecular responses to repeated morphine treatment in male mice. BDPP pre-treatment alters responses for both locomotor sensitization and conditioned place preference. Most notably, polyphenol treatment consistently reduced formation of preference at low dose (5 mg/kg) morphine but enhanced it at high dose (15 mg/kg). In parallel, we performed transcriptomic profiling of the nucleus accumbens, which again showed a dose × polyphenol interaction. We also profiled microbiome composition and function, as polyphenols are metabolized by the microbiome and can act as prebiotics. The profile revealed polyphenol treatment markedly altered microbiome composition and function. Finally, we investigated involvement of the SIRT1 deacetylase, and the role of polyphenol metabolites in behavioral responses. These results demonstrate polyphenols have robust dose-dependent effects on behavioral and physiological responses to morphine and lay the foundation for future translational work.

## Introduction

Opioid use disorder (OUD) is a severe neuropsychiatric condition characterized by cycles of out of control drug intake, persistent use despite negative consequences, and cycles of abstinence and relapse^1,2^. Among substance use disorders, OUDs are particularly problematic given the high propensity for overdose^3,4^. Despite the consequences, rates of pathological OUD have continued to increase over the past decade, with early data suggesting the Covid-19 pandemic has exacerbated the severity of this epidemic^5^. There are currently multiple FDA-approved medications for the treatment of OUD, including opioid agonist replacement therapies and opioid receptor antagonists. While these therapies are effective for some, for many they are ineffective or intolerable^6^. Given the difficulties in treating patients once they have developed an OUD, developing interventions that can prevent the progression to pathological opioid use is of high priority in the field.

Opioid use disorder is widely believed to be the result of a constellation of neuroadaptations that occur through repeated or prolonged drug use^7^. These changes in epigenetic and transcriptional regulation result in altered functional and structural synaptic plasticity^8,9^. More recently, alterations in systemic inflammation and gut-brain signaling have been implicated in the maladaptive behavioral and synaptic plasticity resulting from prolonged opioid exposure^10,11^.

Dietary polyphenols are a class of naturally occurring compounds from botanical sources which are neuroprotective in numerous models of neuropsychiatric disease^12–15^. In a stress-induced model of depression, treatment with polyphenols resulted in increased resilience to the formation of depression-like behaviors^16^. Similar effects have been found in models of neurodegenerative diseases^12,17^. In models of substance use disorders, treatment with dietary polyphenols has been linked to reduced formation of alcohol preference^18–20^ and has been shown to decrease maladaptive neuroplasticity following treatment with psychostimulants^21–23^. While currently there is no published literature on polyphenols and OUD, there is significant literature showing polyphenols can reduce pain in models of pathological pain^24–28^.

Polyphenol compounds exert their neuroprotective effects via myriad mechanisms. They are well known to reduce inflammation and alter redox balance^16,29,30^. Polyphenol compounds, most notably resveratrol, activate the sirtuin family of histone deacetylases, leading to altered epigenetic regulation of gene expression and altered neurobiological and behavioral plasticity^31^. Additionally, polyphenols also exert effects on transcriptional control of gene expression and neuronal function via sirtuin-independent mechanisms^32–34^. Additionally, polyphenols extensively interact with the gut microbiome in ways that are critical for gut-brain signaling^35^.

Here, we use the previously described brain-penetrant bioactive dietary polyphenol preparation (BDPP)^16,32^ to assess how polyphenols may influence the formation of conditioned place preference and locomotor sensitization behaviors and transcriptional regulation in models of OUD. We find that pre-treatment with BDPP can reduce the formation of morphine locomotor sensitization and conditioned place preference. Additionally, we find that treatment with polyphenols interacts with morphine to alter the composition of the gut microbiome and the transcriptome in the nucleus accumbens. Taken together, these findings suggest translational potential for polyphenols in reducing the formation of maladaptive responses to repeated opioid exposure.

## Materials and methods

### Animals

Male C57BL/6J mice (7–9 weeks old, Jackson Laboratories) were group-housed (4–5 mice/cage) in a humidity and temperature-controlled colony room on a 12/12 h light–dark cycle (lights on at 7:00am). Drink solutions and food were available ad libitum throughout the entirety of all experiments. All animal procedures were approved by the Mount Sinai Institutional Animal Care and Use Comittee and all procedures conformed to the “Guide for the Care and Use of Laboratory Animals” (National Research Council 2010). All studies are reported in accordance with the ARRIVE guidelines.

### Preparation and delivery of dietary polyphenols

Cages were randomly assigned to control or BDPP treatment. BDPP treatment consisted of 100 ml of Concord (Welch’s grape juice), 0.4 g Grape Seed Polyphenolic Extract (GSPE) (Healthy Origins #57,914), and 0.4 g resveratrol (Bulk supplements #SKU RES100GC) in 300 ml water as described previously^16,32^. Control animals were provided with water containing matched sucrose content. Mice were treated for 2 weeks prior to the start of experimental procedures and all mice remained on their drink solutions until the conclusion of the studies.

### Morphine

Morphine sulfate was provided by the NIDA drug supply program from National Institute on Drug Abuse and was diluted in saline and injected subcutaneously.

### Locomotor sensitization

Locomotor sensitization was performed largely as described previously^36^. The locomotor arena consisted of a frame crossed with infrared beams in the x and y dimensions. Clean, empty rat cages were placed within this frame to contain the mice while simultaneously allowing penetration of the infrared beams. Animals could freely move throughout the space and infrared beam breaks were counted to determine locomotor activity. Control or BDPP treated mice were injected with saline, 5 mg/kg, or 15 mg/kg morphine and their activity monitored for the subsequent 45 min. For “challenge injection” experiments mice were tested for five days before being returned to their home cages for ten days of abstinence. Ten days later they were given a morphine challenge injection of the same dose as the initial injections and were placed back into the locomotor arena to measure persistence of morphine locomotor sensitization.

### Morphine conditioned place preference

Mice from Control and BDPP treatment groups were injected with 2.5, 5, or 15 mg/kg morphine and underwent morphine conditioned place preference (CPP) largely as described previously^36^. Each CPP apparatus consists of 3 distinct chambers: a middle, small entry chamber and two larger conditioning chambers on either side of the entry. Conditioning chambers were distinct in wall color and floor texture. The left end chamber had gray walls and a large grid floor, and the right end chamber had black and white striped walls and a small grid floor. Conditioned place preference occurred over 5 days: day 1 was a pre-test, days 2–4 were conditioning days, and the fifth day was the post test. On pre-test day, mice were placed into the center chamber of the apparatus and were allowed to explore all 3 chambers for 20 min. Time spent in each chamber was recorded; mice spending > 70% of their time in one chamber were excluded. Mice were assigned to their morphine-paired chamber using an unbiased approach such that group preference on pre-test was as close to zero as possible. On conditioning days, mice were injected with saline subcutaneously in the morning and confined to one end chamber and injected with either 2.5, 5, or 15 mg/kg morphine and confined in the opposite end chamber in the afternoon. Both morning and afternoon conditioning sessions lasted 45 min. On test day, mice were again allowed to explore all 3 chambers of the apparatus for 20 min, as described above for pre-test. Place preference score was calculated as: time spent in the morphine chamber on day 5—time spent day 1 (Test–PreTest).

### Withdrawal study CPP

Mice were injected with 5 mg/kg morphine in their home cages for 5 consecutive days. They were then given control or BDPP treatment for two-weeks during drug abstinence. Mice then underwent CPP as described above to measure the effect of morphine pre-exposure on formation of preference.

### RNA-sequencing of NAc

Control or BDPP treated mice underwent CPP paradigm injected with saline, 5 mg/kg, or 15 mg/kg morphine and were rapidly decapitated 1 h post-test session of CPP. The Nucleus Accumbens (NAc) was flash frozen on dry ice. RNA from frozen NAc punches was isolated using RNeasy kits (Qiagen-#74,106) with on-column DNAase digestion (#79,254) per manufacturer’s protocol. The integrity and purity of total RNA were assessed using Agilent Bioanalyzer and OD260/280 using Nanodrop. The RNA sequencing library was generated using NEBNext Ultra II RNA library Prep Kit for Illumina using manufacturer’s instructions (New England Biolabs, Ipswich, MA, USA). The sequencing library was validated on the Agilent TapeStation (Agilent Technologies, Palo Alto, CA, USA), and quantified by using Qubit 2.0 Fluorometer (ThermoFisher Scientific, Waltham, MA, USA) as well as by quantitative PCR (KAPA Biosystems, Wilmington, MA, USA). The libraries were then multiplexed and sequenced on an Illumina HiSeq 4000 instrument using a 2 × 150 bp Paired End (PE) configuration according to manufacturer’s instructions. Image analysis and base calling were conducted by the HiSeq Control Software (HCS). Raw sequence data (.bcl files) generated from Illumina HiSeq was converted into fastq files and de-multiplexed using Illumina bcl2fastq 2.20 software. One mismatch was allowed for index sequence identification.

### RNA-seq data analysis

The raw RNA-Seq reads (Fastq files) for each sample were aligned and read counts generated using the cloud-based BioJupies software package using the default settings^37^. Raw count matrices were then analyzed using the Network Analyst Software package with the variance filter set to 5% and the low abundance filter set to 2^38,39^. Data were normalized to Log2 counts per million. Differential gene expression analysis was then performed using Deseq2. Statistical significance was set at a threshold of FDR-corrected *p* < 0.2 except as described. For pathway and transcription factor analysis all genes marked as “predicted gene”, “pseudogene”, Riken gene, and ribosomal protein encoding genes were removed prior to analysis. Identification of significantly enriched gene ontologies was performed using the g:Profiler analysis package^40^. For identification of enrichment of predicted transcription factor activity we utilized the Enrichr software package querying the Chea and ENCODE datasets to identify targets based on publicly available ChIP-seq datasets^41,42^. Volcano plots were made using GraphPad Prism. Group Ns for this Experiment: H_2_O/Sal 6; BDPP/Sal 4; H_2_O/5 mg 5; BDPP/5 mg 6; H_2_O/15 mg 7; BDPP/15 mg 6.

### 16 s-sequencing of cecal content

Control or BDPP treated mice underwent CPP paradigm injected with 5 mg/kg or 15 mg/kg morphine and were euthanized 24 h post-test session. The caecum was rapidly dissected, contents flash frozen on dry ice and stored at -80C. Bacterial genomic DNA was isolated from frozen cecal samples using the DNeasy PowerSoil Pro kit (Qiagen) according to standard protocols with a modification that included an extended bead beating step. Extracted DNAs were checked for quality and quantity by spectrophotometric measurements with NanoDrop (ThermoFisher Scientific Inc). PCR amplification was subsequently achieved using primers 341F (5′-CCTACGGGNGGCWGCAG-3′) and 805R (5′-GACTACHVGGGTATCTAATCC-3′) targeting regions (V3–V4) of the 16S rRNA gene. The libraries were sequenced using illumina NovaSeq (2 × 250 bp paired-end) platform using standard parameters.

#### 16 s-sequencing data analysis

Amplicons were trimmed, merged using FLASH^43^ and chimera filtered using Vsearch (v2.3.4). Sequences with ≥ 97% similarity were assigned to the same operational taxonomic units (OTUs). Representative sequences were chosen for each OTU, followed by taxonomic assignment using the RDP (Ribosomal Database Project) classifier. The differences of the dominant species in different groups and multiple sequence alignment were conducted by mafft software (v7.310). OTU abundance information was used to determine alpha diversity as the Chao1 metric. Principle coordinates analysis plots were generated using the Unifrac distance as an assessment of beta diversity, both using the Quantitative Insights Into Microbial Ecology (QIIME) package v1.8.0^44^. Statistical significance for alpha diversity was analyzed using a 2 × 2 between subjects ANOVA with morphine dose and drink solution provided as fixed factors. At the phylum and genus level, any bacterial groups with mean expression levels ≥ 0.05% abundance and a Wilcoxon’s FDR corrected *p* value < 0.2 were considered statistically significant. There was an N of 8/group for all groups assessed.

#### Microbial functional profile prediction and LEfSe methods

Predicted functional profiles of bacterial groups were determined using Phylogenetic Investigation of Communities by Reconstruction of Unobserved States (PICRUSt2) bioinformatics software package as published^45^. Firstly, the OTU table produced within QIIME was normalized for multiple 16 s RNA copy numbers. Then the obtained normalized OTU table was placed into a reference tree in order to obtain the Kyoto Encylopedia of Genes and Genomes (KEGG) orthologs (KO). In order to assess the functional pathways, including enzymatic pathways affected by microbiome changes due to BDPP treatment and morphine dose, sequence counts of all KO pathways for each sample were converted to mean proportion percent using the formula: (sequence count for specific KO pathway / total sequence count of all pathways × 100). Subsequently a two-tailed students *t*-test and FDR < 0.01 was applied to all pathways. K identifiers of KO pathways which met this significance criteria were then uploaded into the KEGG database www.genome.jp/kegg/ko.html to identify mapped cellular functions^46–48^.

OTU tables generated were further subjected to Linear discriminant analysis (LDA) effect size (LEfSe) to calculate the taxa that best discriminated between control and BDPP treatment groups. Specifically, the non-parametric factorial Kruskal–Wallis (KW) sum-rank test was used to detect taxa with significant differential abundance among groups. Taxa consistency was then investigated using a set of pairwise tests among subclasses with the (unpaired) Wilcoxon rank-sum test. Finally, LEfSe uses LDA to estimate the effect size of each differentially abundant taxa. Taxa that reached a linear discriminant analysis score (log10) > 3.0 and *p* < 0.05 are reported. Taxa that discriminating between treatment groups are visualized on taxonomic trees called Cladograms. Cladograms show how the taxa that discriminate between the various treatment groups are related to each other in terms of taxonomy and ontologies of functional pathways.

#### SIRT1 inhibitor study

To assess the role of SIRT1 in mediating BDPP effects, control or BDPP treated mice were anesthetized with a combination of ketamine (100 mg/kg) and xylazine (10 mg/kg) and surgically implanted with bilateral cannula targeting the NAc under stereotactic guidance. The coordinates from bregma were: anteroposterior 1.5 mm, mediolateral 1.0 mm, dorsoventral 4.5 mm. Animals were allowed to recover from surgeries for one week prior to undergoing CPP as described above using 5 mg/kg morphine. Animals were maintained on drink solutions throughout. On each of the first four days of CPP 0.05 mM of EX-527 (Sigma-Aldrich) a SIRT1 specific inhibitor or vehicle (DMSO) was infused into the NAc over 5 min immediately after the morphine session.

#### DHCA/Mal-Gluc treatment study

Mice were randomly divided into two groups: control group provided with standard drinking water, and a group treated with a mixture of dihydrocaffeic acid (DHCA—5 mg/kg-BW/day) and malvidin-3′-*O*-glucoside (Mal-gluc 0.5 µg/kg/day), delivered through their drinking water as previously described^16^, both treatments starting 2 weeks prior to CPP using 5 mg/kg and 15 mg/kg morphine.

#### Statistical analysis, figures, and data availability

All behavioral analyses were performed using GraphPad Prism with two-way ANOVAs with repeated measures as appropriate for 2 × 2 experiments, with Fisher’s exact tests being utilized as post-hoc tests. Post-hoc testing was only performed for comparisons in which the ANOVA produced a significant main effect or interaction so as to reduce spurious comparisons. Two-tailed T-tests were used for pairwise comparisons. 16S and RNA sequencing data were analyzed as detailed in Supplemental Methods. Graphs of all figures were created in Graphpad Prism and R. Experimental timelines were generated in BioRender with full permission to publish. Raw data from RNA-sequencing analyses are available on GEO with accession number GSE224937. Permission for publication of the KEGG pathways was kindly provided by the Kanehisa laboratories.

## Results

### Controls for polyphenol treatment

**Figure 1:**
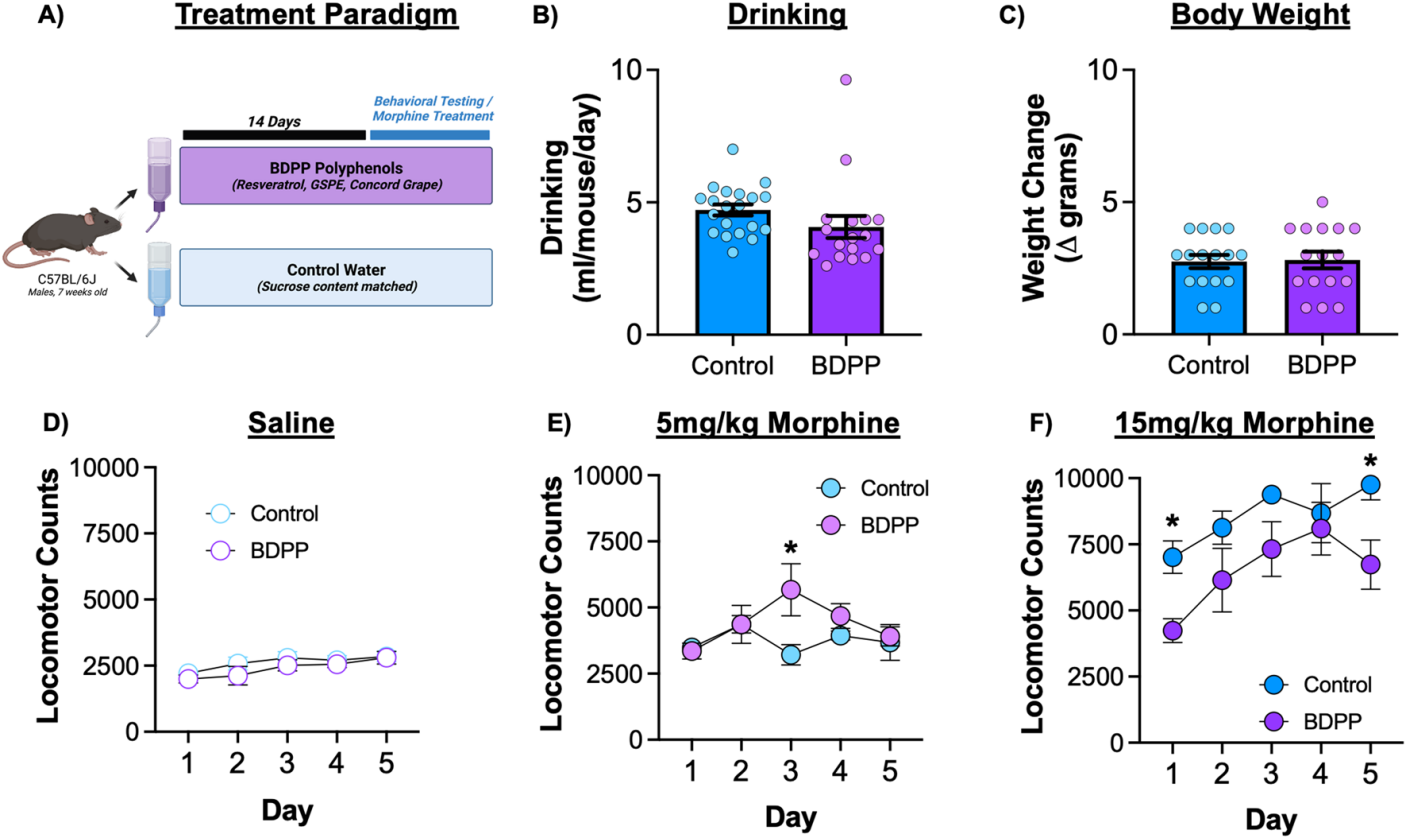
Baseline effects of BDPP polyphenols and locomotor response to morphine. (**A**) Graphical timeline of the associated studies. (**B**) To ensure that treated water did not affect drinking we measured consumption in a subset of animals during the first two weeks. (**C**) Body weight change over the course of the experiment was also measured. (**D**) Repeated injections of saline did not affect locomotor activity in either treatment group. (**E**) Repeated 5 mg/kg injections of morphine resulted in a significant time x treatment interaction with polyphenol animals having increased locomotion on day 3. (**F**) Polyphenol treatment resulted in decreased development of locomotor sensitization to 15 mg/kg morphine. Data presented as means ± SEM. *p < 0.05; **p < 0.01. N: For panels B&C = 16–20/group; For panels D-F = 6–8/group.

For most studies C57BL/6J mice were treated with BDPP polyphenol cocktail or control water in their home cage two weeks prior to the start of experimentation and remained on these drink solutions throughout duration of the study (Fig. 1A). A subset of mice were monitored for water intake and bodyweight change as indicators of overall health. We found no significant difference in drink intake (Fig. 1B—t_(35)_ = 1.45, *p* = 0.16) or bodyweight change between the groups (Fig. 1C—t_(30)_ = 0.15, *p* = 0.88).

### Locomotor sensitization

To determine the effects of BDPP supplementation on behavioral response to opioids, we measured activity in a locomotor sensitization paradigm. As a control we tested all animals with five daily repeated injections of saline. There was no effect of BDPP treatment (Fig. 1D—two-way RM-ANOVA: F_(1,14)_ = 1.30; *p* = 0.27), a modest effect of time (F_(4,56)_ = 4.83; *p* = 0.002) but no significant interaction (F_(4,56)_ = 0.36; *p* = 0.83). At the lower 5 mg/kg dose of morphine there was no effect of treatment (F_(1,11)_ = 1.39; *p* = 0.26) or time (F_(4,44)_ = 2.20; *p* = 0.085). However, there was a significant treatment by time interaction (F_(4,44)_ = 3.15; *p* = 0.02), with post-hoc testing demonstrating significant increase in locomotor response in the BDPP group on Day 3 (Fig. 1E). Sensitization effects at 15 mg/kg dose of morphine resulted in effects of both treatment (Fig. 1F—F_(1,10)_ = 12.49; *p* = 0.005) and time (F_(4,39)_ = 4.3; *p* = 0.006) with the BDPP group showing reduced locomotor activation in response to acute and repeated morphine. There was no significant time x treatment interaction (F_(4,39)_ = 0.68; *p* = 0.6). Additional details on acute and sensitized locomotor response are available in SupplSupplemental Results and Fig. S1.

### Conditioned place preference

**Figure 2:**
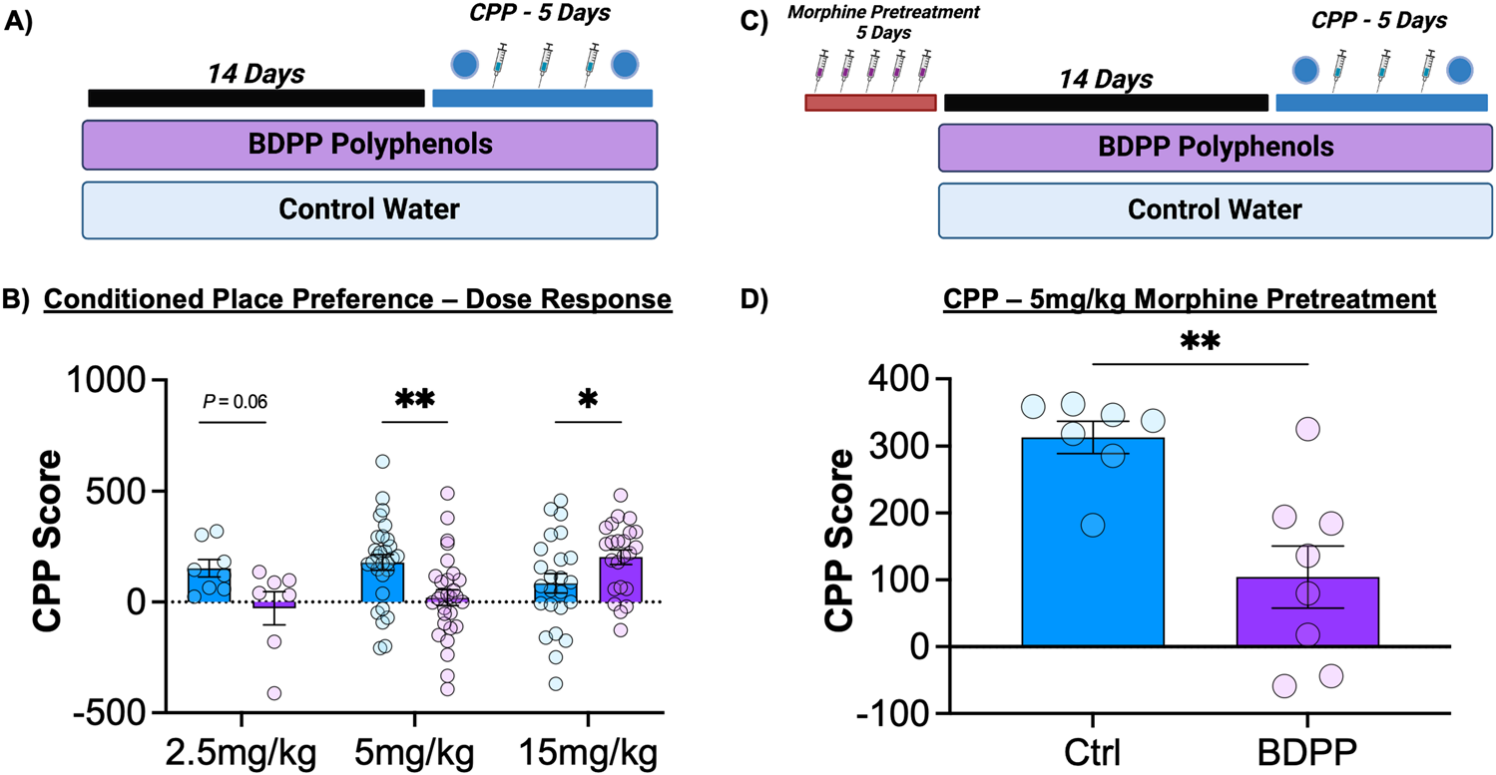
Effects of BDPP Polyphenols on formation of morphine conditioned place preference. (**A**) After two weeks of BDPP or control pretreatment, mice were tested on a conditioned place preference assay using a five-day protocol. (**B**) CPP results across a dose range showed a robust dose x treatment interaction with BDPP-treated mice showing decreased preferences at lower doses of morphine, but increased preference for the higher 15 mg/kg dose. (**C**) In a subsequent experiment, mice were pretreated with morphine prior to polyphenol treatment and CPP testing. (**D**) While morphine pretreatment enhanced subsequent formation of CPP, BDPP polyphenol pretreatment still resulted in reduced formation of preference at 5 mg/kg morphine. Data presented as means ± SEM. *p < 0.05; **p < 0.01. N = 7–29/group with individual points on graphs.

While locomotor sensitization is an important marker of plasticity in response to drugs of abuse, it has little specificity for assessing rewarding drug effects. To test how BDPP pre-treatment modulated the rewarding effects of morphine we performed CPP testing for morphine (Fig. 2A). When assessed by two-way ANOVA we find that there was a trend but no effect of BDPP treatment (F_(1,114)_ = 3.31; *p* = 0.07), and no effect of morphine dose (F_(2,114)_ = 1.35; *p* = 0.26). However, there was a BDPP x dose interaction (F_(2,114)_ = 8.17; *p* = 0.0005)—which is likely responsible for abrogating the main effect of dose. On post-hoc testing, BDPP treatment led to a marked decrease in preference at the intermediate 5 mg/kg dose (Fig. 2B middle—*p* = 0.002), but a significant increase in CPP at 15 mg/kg (Fig. 2B right—*p* = 0.03).

As prior treatment with opioids can lead to potentiation of future behavioral response in subsequent place preference^49^, we then tested the effect of BDPP on CPP for intermediate dose morphine using an opioid re-exposure model. Mice received injections of 5 mg/kg morphine for five days prior to the start of polyphenol treatment, they then underwent the normal BDPP treatment and CPP procedure (Fig. 2C). As predicted, the pre-treatment with morphine potentiated morphine CPP compared to the standard regimen (Fig. S2). However, even when mice had been pre-treated with morphine prior to polyphenols, the two weeks of BDPP again resulted in a reduction of CPP (Fig. 2D—t_(13)_ = 3.81; *p* = 0.002).

### RNA-sequencing of the nucleus accumbens

**Figure 3:**
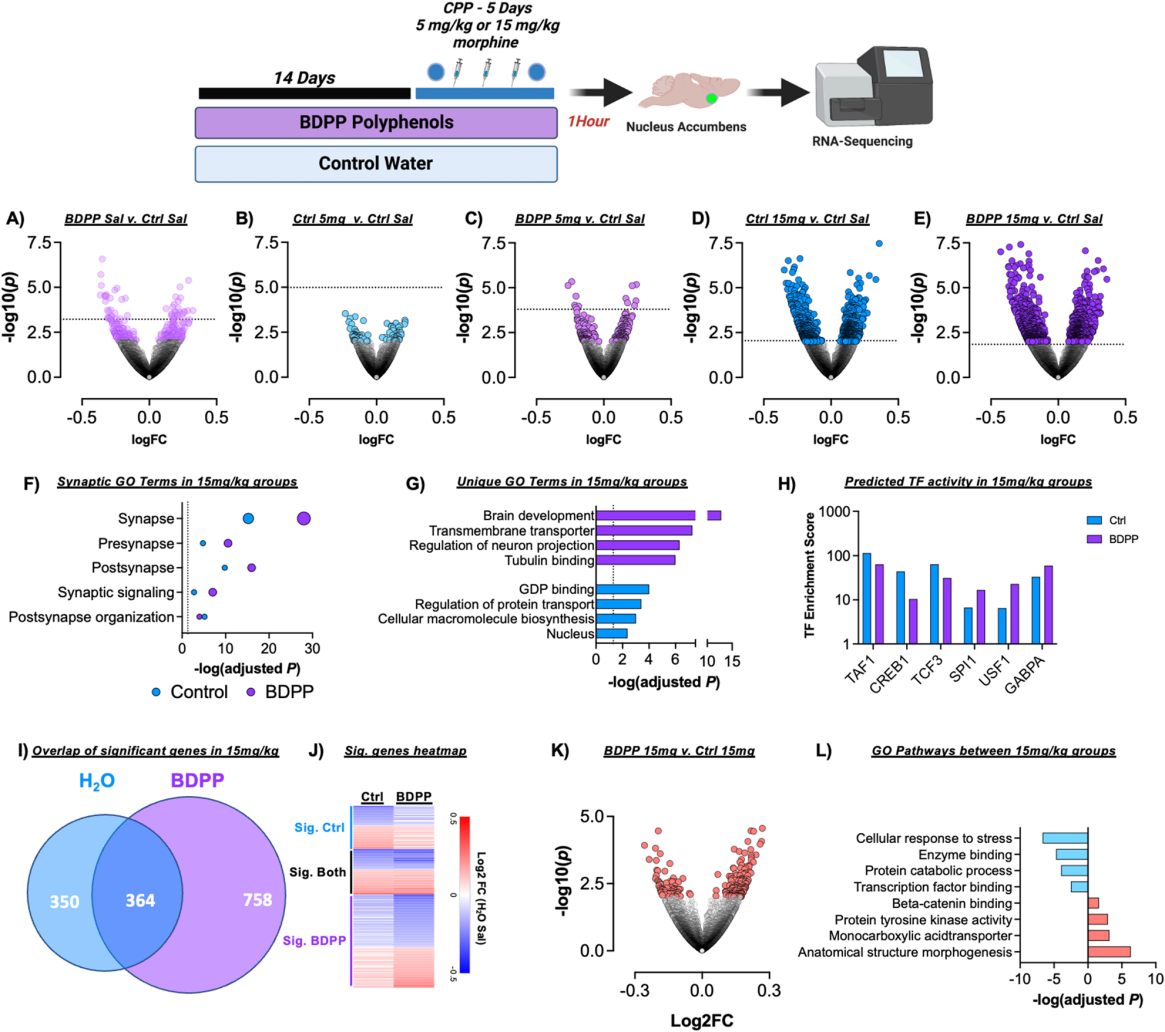
Effects of BDPP polyphenol treatment on transcriptional response to morphine in the nucleus accumbens. (Top) Timeline for sequencing experiments. Mice were pretreated for two weeks prior to CPP training and were sacrificed one hour after the post test session. (**A**–**E**) Volcano plots of all treatment groups relative to Control Saline group. (**F**) Gene ontology analysis of synapse related genes in H2O and BDPP 15 mg/kg groups. (**G**) GO terms uniquely regulated in BDPP 15 mg/kg and Ctrl 15 mg/kg groups compared to Ctrl Saline. (**H**) Transcription factor enrichment analysis of top enriched treatment groups in Ctrl and BDPP 15 mg/kg groups. (**I**) Venn diagram off all genes statistically significant in Ctrl and BDPP 15 mg/kg groups relative to Ctrl Saline. (**J**) Heatmap of fold change expression of all genes from the previous panel. (**K**) Volcano plot of all significantly different genes between the two morphine 15 mg/kg groups. Fold change is relative to Ctrl 15 mg/kg. (**L**) Gene ontology analysis of top terms regulated up or down between the two 15 mg/kg morphine groups.

Substance use disorders and behavioral adaptations to drugs of abuse are dependent on alterations in regulation of gene expression^8^. Additionally, dietary polyphenols, alter gene expression in the brain^16,50^. To better understand how BDPP treatment alters neurobiological response to morphine, we performed RNA-sequencing of the nucleus NAc—a key structure in driving behavioral response to opioids^51^. For these experiments, animals underwent CPP as normal, and a control group received saline injections during each session (Fig. 3—top).

To obtain a broad understanding of differential gene expression, we created volcano plots of all groups compared to Control-Saline (Fig. 3A–E, colored circles *p* < 0.01). As expected, the most robust effects on gene expression were seen with the higher 15 mg/kg dose of morphine (Fig. 3D,E). Interestingly, treatment with BDPP resulted in more significantly regulated genes at both doses, and treatment with BDPP alone resulted in more significantly regulated genes than mice treated with water or BDPP in combination with 5 mg/kg morphine (Fig. 3A–C). Full differential gene lists for each pairwise comparison available as Supplemental Tables 1–5. Additionally, we examined genes with a nominal *p* value < 0.01 in BDPP groups compared to control saline at both 5 mg/kg and 15 mg/kg doses. Interestingly, we find that BDPP treatment did not change the directionality of expression in response to morphine for any of these genes, the fold change in the 15 mg/kg group was larger for all (Fig. S3). This is consistent with previous literature that BDPP treatment has a modulatory effect on gene expression, but does not override effects of robust external stimuli^24,32^.

We then examined changes in genes that met statistical significance (FDR *p* < 0.2) (Fig. 3A–E dotted line indicates the nominal *p* value at which this threshold is met). As there were more significantly regulated genes in the 15 mg/kg morphine groups, (Fig. 3D,E) detailed pathway analysis was conducted for the 15 mg/kg groups. Both control and BDPP 15 mg/kg morphine treated mice had significant enrichment of genes related to the synapse, both pre and post synaptic function, synaptic signaling, and postsynaptic organization when compared to control Saline (Fig. 3F; Full GO Tables S6, S7). In all these pathways enrichment was more robust in the BDPP 15 mg/kg morphine animals.

Additionally, there were numerous uniquely enriched pathways in the two groups. BDPP 15 mg/kg mice showed significant enhancement of brain development, transmembrane transporters, and tubulin binding (Fig. 3G), while control 15 mg/kg mice had enrichment of GDP-binding, macromolecule biosynthesis, and nuclear genes. Next, the Enrichr software package was used to analyze differences in transcription factor involvement between control 15 mg/kg and BDPP 15 mg/kg morphine groups. We find that treatment with control or BDPP resulted in differential recruitment of transcription factors (Fig. 3H—note log10 y-axis; full Enrichr results Table S8).

Of the 1472 genes that were regulated compared to control saline between the two 15 mg/kg morphine groups, 364 were significantly regulated in both groups, while 350 uniquely changed in control and 758 uniquely changed in BDPP (Fig. 3I). When all of these genes were visualized as a heatmap examining fold change relative to control Saline animals, nearly all genes change in the same direction (Fig. 3J)—but not necessarily statistically significant in both.

Finally, both 15 mg/kg morphine groups were compared to examine how the addition of BDPP pre-treatment affected gene expression when compared directly to other morphine paired animals. Figure 3K shows a volcano plot depicting the 205 genes that were statistically significant at a *p* < 0.01 threshold (67 downregulated, 139 upregulated) between control 15 mg/kg and BDPP 15 mg/kg. Gene ontology analysis was performed separately on the up and downregulated gene lists. BDPP 15 mg/kg mice showed decreased expression in pathways related to cellular response to stress and transcription factor binding among others, A and increases in pathways related to anatomical structure, morphogenesis, and protein tyrosine kinase activity (Fig. 3L—Full table: Table S9).

### 16 s sequencing of cecal content

**Figure 4:**
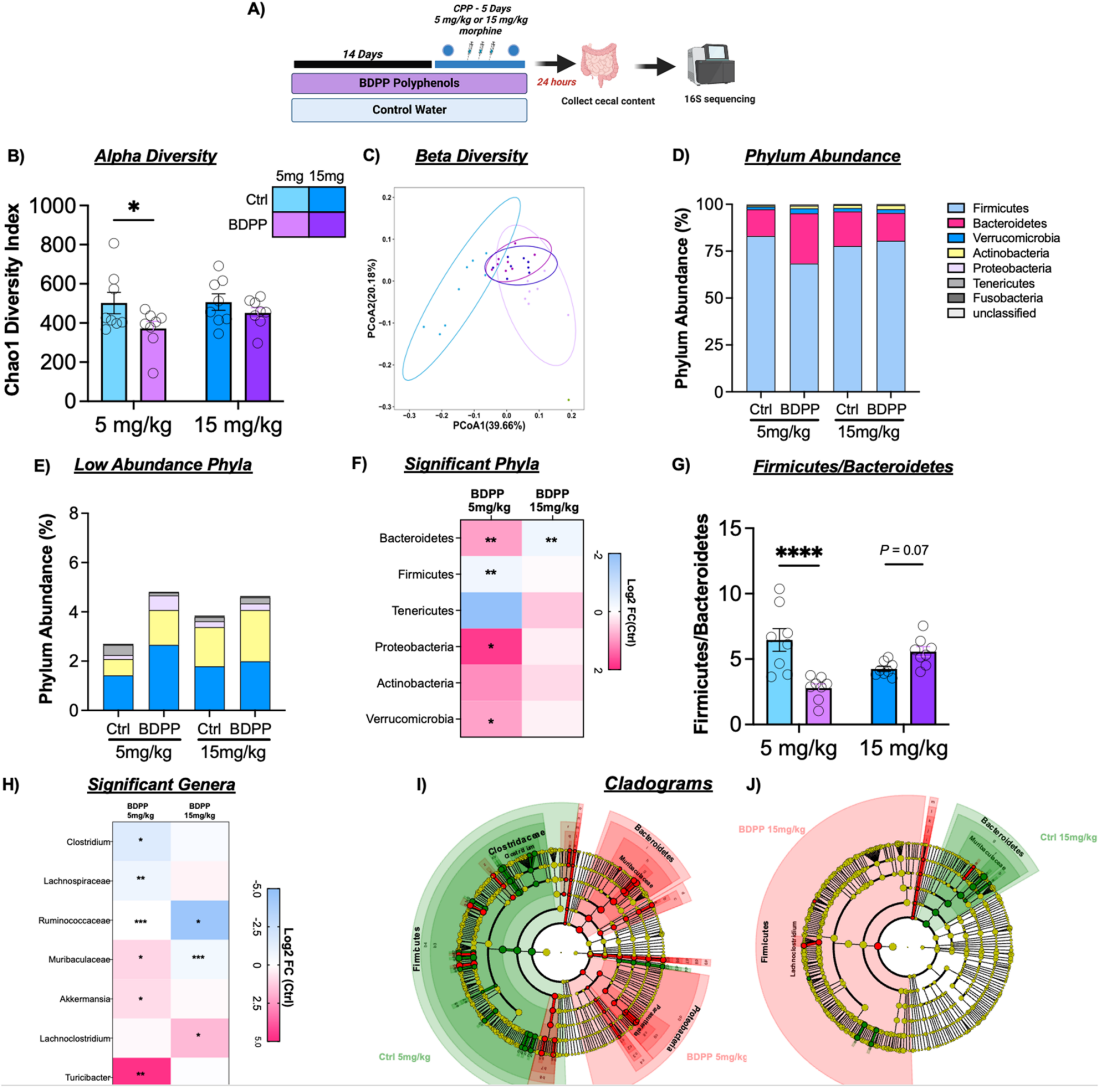
Effects of BDPP treatment on cecal microbial composition in morphine treated mice. (**A**) Timeline for 16 s sequencing experiment. Mice were pre-treated for two weeks prior to CPP training and were sacrificed 24 h after the post test session. (**B**) Alpha diversity of the gut microbiomes were calculated using Chao1 diversity metric, and shows reduced Alpha diversity in 5 mg/kg BDPP treated mice (**C**) Beta diversity measured using the unweighted Unifrac distance metrics and shows BDPP treated mice possess a unique microbiome from control treated mice in 5 mg/kg morphine group, but overlap in 15 mg/kg morphine group (**D**) Stacked bar chart showing the relative phylum abundance in mice from all treatment groups, each phyla represented in a different color (**E**) Stacked bar chart showing phyla expression levels of the low abundance phyla (this chart corresponds to the top section of the graphs from panel **D**). (**F**) Heatmap showing changes in phylum diversities with BDPP treatment relative to controls at each dose. Asterisks represent *p* values from Wilcoxon text. (**G**) BDPP treated mice show a significant decrease in Firmicutes to Bacteroidetes Ratio compared to controls in the 5 mg/kg morphine treatment group (**H**) Heatmap displaying the log2 Fold Change (FC) of selected altered bacterial genera in BDPP treated animals relative to respective control counterparts in 5 mg/kg and 15 mg/kg morphine group. Asterisks represent *p* values from Wilcoxon text. (**I**) Cladogram representing taxonomic biomarkers characterizing the differences between BDPP and control treated mice in the 5 mg/kg morphine group (**J**) Cladogram with taxonomic biomarkers characterizing the differences between BDPP and control treated mice in the 5 mg/kg and 15 mg/kg morphine groups (Full Key for figures** I** and** J** found in Tables S13). *p < 0.05 **p < 0.01 ***p < 0.001. N = 8/group.

Given the interactions between dietary polyphenols and the microbiome^52,53^, effects of BDPP treatment and interactions with morphine were assessed (Fig. 4A). Analysis of alpha diversity, which calculates richness and evenness of microbial species, using the Chao1 metric^54^ showed a significant effect of BDPP (Fig. 4B—F_(1,28)_ = 5.06; *p* = 0.03), no effect of morphine dose (F_(1,28)_ = 1.05; *p* = 0.31) and no significant BDPP x morphine dose interaction (F_(1,28)_ = 0.82; *p* = 0.37). Post-hoc testing showed BDPP treatment led to significantly decreased alpha diversity only in the 5 mg/kg morphine group (Fig. 4B—left—*p* = 0.03). Next β‐diversity, a measure of between‐subject diversity, was assessed which revealed a clear separation between control and BDPP treated animals in the 5 mg/kg morphine group. However, no separation between water control and BDPP treated animals was observed in the 15 mg/kg morphine group (Fig. 4C).

Next, the effect of BDPP treatment on phylum abundance was examined. In the qualitative stacked bar plots in Fig. 4D,E, we see that BDPP results in shifts in phylum expression in the 5 mg/kg group in the highly abundant Bacteroidetes and Firmicutes phyla (Fig. 4D—pink and blue bars), as well as in the less abundant phyla (Fig. 4E). Given that in mammals Bacteroidetes and Firmicutes make up ~ 90% of total bacteria found in the gut, the relative abundance of these phyla has thus been tied to the health of the microbiome^55^. In the 5 mg/kg morphine group, BDPP treatment significantly decreased the abundance of bacteria from the Firmicutes phylum (Fig. 4F left—Wilcoxon rank-sum test: *p* = 0.002) and significantly increased the abundance of Bacteroidetes (*p* = 0.002). In the 15 mg/kg morphine treatment group, the opposite effect is observed where BDPP treatment resulted in a trend towards an increase in the abundance of Firmicutes (Fig. 4F right—Wilcoxon rank-sum test: *p* = 0.046; q = 0.23) and a significant decrease in Bacteroidetes *(p* = 0.006) compared to control animals. Full list of phyla changes and statistics in Table S10.

Shifts in the ratio of the Bacteroidetes and Firmicutes phyla can be an important marker of microbiome health and stability. When the ratio of these two major phyla is compared, there was a modest main effect of BDPP treatment (Fig. 4G—F_(1,28)_ = 5.34; *p* = 0.03), and no effect of morphine dose (F_(1,28)_ = 0.30; *p* = 0.59). There was, however, a robust dose x treatment interaction (F_(1,28)_ = 23.91; *p* < 0.0001). Post-hoc testing comparing the treatment groups within each dose showed a significant effect of BDPP at 5 mg/kg (Fig. 4G left—*p* < 0.0001).

We next examined phylogenetic changes down to the genus level. In the 5 mg/kg group, bacteria belonging to the genus *Clostridium* were significantly reduced in BDPP treated animals (Fig. 4H—Wilcoxon, *p* = 0.02; Full Table S11) however this reduction was not observed in 15 mg/kg morphine treated animals (*p* = 0.17). In the 5 mg/kg morphine group BDPP treatment resulted in a significant increase in a genus of *Muribaculaceae* (*p* = 0.01), but this same genus was decreased at 15 mg/kg morphine (*p* = 0.0008). Effects of BDPP treatment on the gut microbiome at the genus level are observed more clearly in the low dose morphine group, with significant increases in beneficial genera *Parasutteralla* (*p* = 0.01) and *Akkermensia* (*p* = 0.02). This is in line with the body of literature linking polyphenol treatment with increases in beneficial bacteria^56,57^*.*

Next, LEfSe analysis was used to determine treatment-defining expression patterns^58^. Full differential bacterial group lists for each pairwise comparison available as Fig. S4A and B. Cladograms show that at 5 mg/kg morphine, control treated mice characterized by formation of CPP, display a Firmicutes/Clostridium enterotype (Fig. 4I green) while BDPP treated mice who did not form CPP, display a contrasting Bacteroidetes/*Muribibaculaceae* and Proteobacteria/*Parasutterella* enterotype (Fig. 4I—red). Interestingly at 15 mg/kg morphine, the control group who did not form CPP, display a Bacteroidetes/*Muribibaculaceae* enterotype while BDPP treated mice characterized by formation of CPP, display a Firmicutes/*Lachnoclostridium* enterotype.

**Figure 5:**
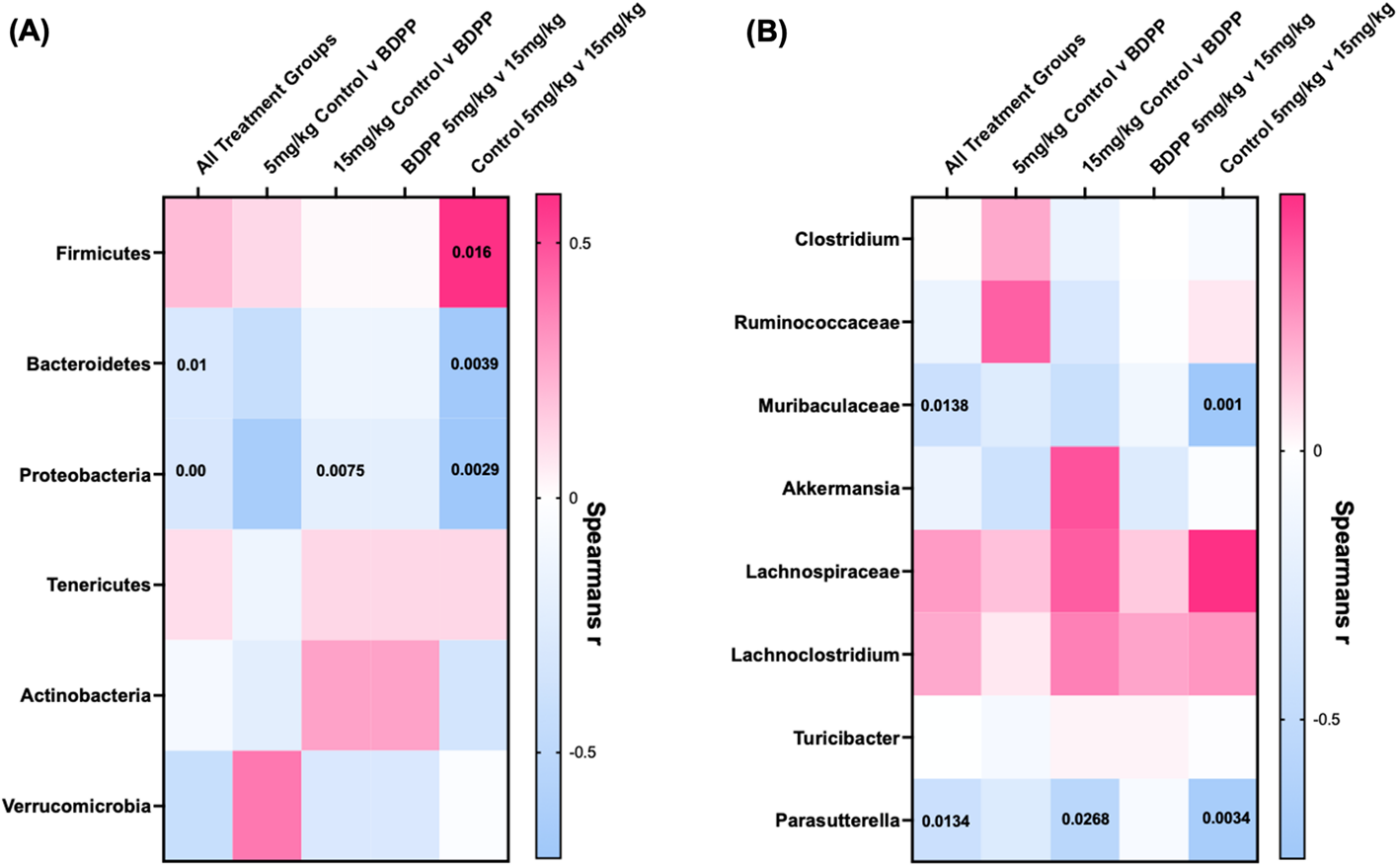
Correlations between Phyla and Genus Total Abundance and CPP Score. Mice that were pre-treated with BDPP for two weeks followed by CPP testing had cecal content collected 24 h after test day for 16 s analysis (**A**) Correlation heatmap of select phyla (columns) with CPP preference, across all treatment groups or within individual treatment groups (rows). Exact r values for each phylum and exact *p* values are available in Supplementary Table S14. (**B**) Correlation heatmap of individual select genus (columns) with CPP preference across all treatment groups or within individual treatment groups (rows). Exact r values for each genus and exact *p* values are available in Supplementary Table S15.

Finally, LEfSE findings were further supported by a series of correlations between select bacterial phyla/genus (significantly changed by treatment) and scores from the CPP paradigm. Here we see Bacteroidetes and Proteobacteria showed a significant negative linear correlation with CPP across all treatment groups (Fig. 5A Left column Spearman* r* = − 0.043, *p* = 0.01 and Spearman* r* = − 0.492, *p* = 0.00 respectively). Indicating a higher abundance of Bacteroidetes and Proteobacteria may be protective against morphine CPP in line with LEfSE results. While the abundance of Firmicutes did not show a significant correlation with CPP across all treatment groups, there was a strong trend towards a positive linear correlation (Fig. 5A Top left Spearman* r* =  − 0.34, *p* = 0.05). Suggesting a higher abundance of Firmicutes may contribute to the development of morphine CPP. At the genus level, we find *Muribibaculaceae* and *Parasutterella* negatively correlate with CPP score when comparing all treatment groups (Fig. 5B Left column Spearman* r* = − 0.431, *p* = 0.014 and Spearman* r* = − 0.433, *p* = 0.013 respectively). Together 16 s analysis results further reinforce morphine dose by BDPP treatment effects and indicate protection from morphine CPP may be driven by a Bacteroidetes/*Muribibaculaceae* and Proteobacteria/*Parasutterella* enterotype while formation of morphine CPP is driven by a Firmicutes/ bacteria belonging to *clostridium* genus enterotype.

### Mechanistic interrogation of polyphenol effects

**Figure 6:**
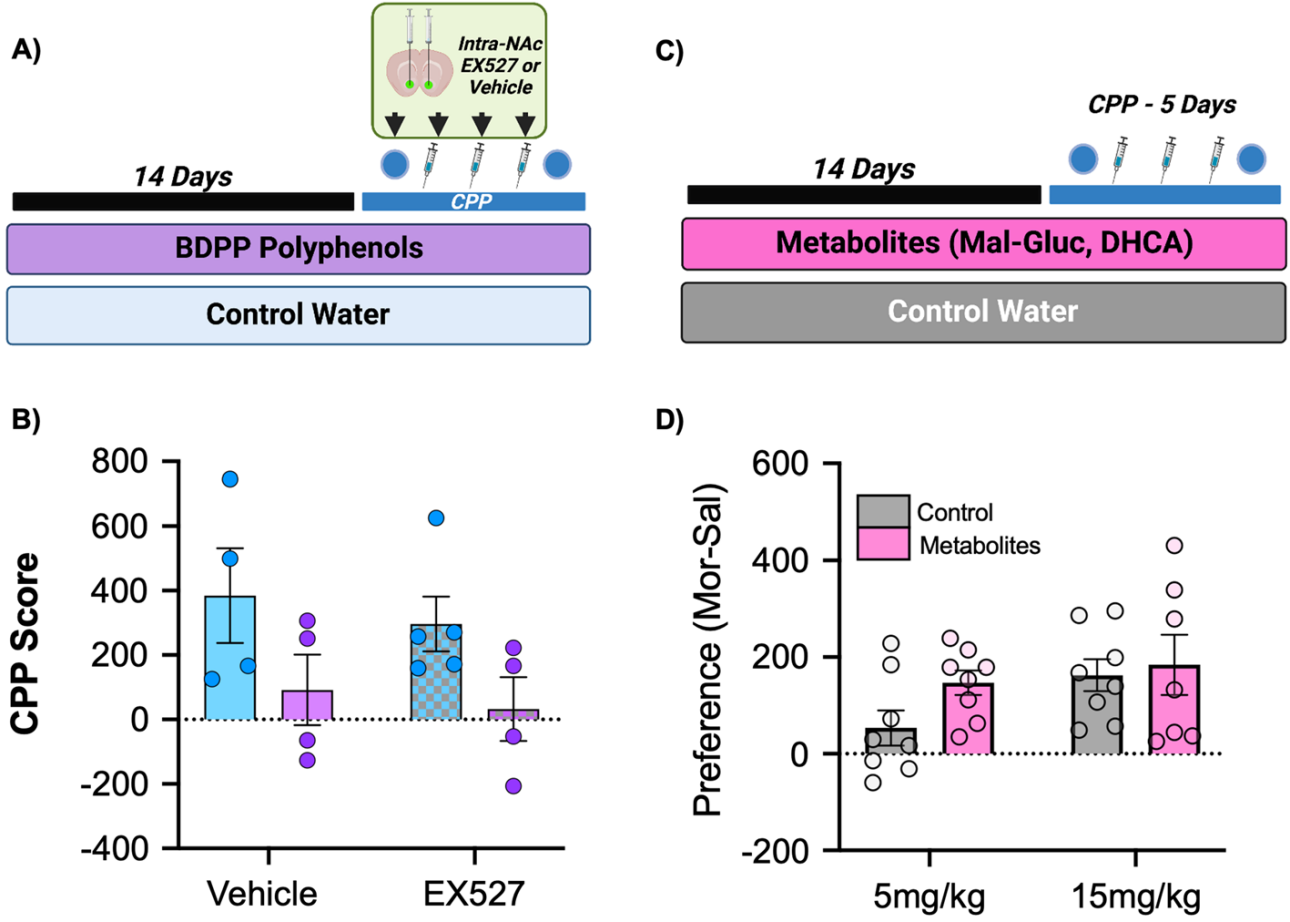
Mechanistic studies of polyphenol effects. (**A**) To test the contribution of SIRT1 activation to the behavioral effects of BDPP treatment a SIRT1 inhibitor was infused into the NAc during CPP. (**B**) CPP at 5 mg/kg morphine again showed a main effect of BDPP treatment, but no effect of SIRT1 inhibition. (**C**) Effects of treatment with two key metabolites from the BDPP cocktail was assessed. (**D**) Treatment with BDPP metabolites did not result in significant changes in morphine preference at either dose. N = 4–8/group with individual points on graphs.

We finally performed two targeted experiments to refine our mechanistic understanding of how BDPP treatment alters behavioral response to morphine. For the first, we based our experiment on the hypothesis that resveratrol and other polyphenols are activators of the SIRT1 lysine deacetylase—which has been shown to be important for behavioral response to opioids^59^. For these experiments, mice were treated with control water or BDPP as previous but had the specific SIRT1 inhibitor infused directly into their NAc after each of the first four days of CPP (Fig. 6A). For this experiment we still found a main effect of BDPP treatment (Fig. 6B—F_(1,13)_ = 6.38; *p* = 0.025), similar in magnitude and direction to what was seen with BDPP treatment alone (Fig. 2B). However, there was no main effect of EX527 treatment (F_(1,13)_ = 0.44; *p* = 0.52) or significant BDPP x EX527 interaction (F_(1,13)_ = 0.02; *p* = 0.9). This indicates that effects of BDPP on CPP behavior at 5 mg/kg are not mediated by SIRT1 activity in the NAc. While the N was smaller for the initial cohort, the lack of any appreciable effect of our intervention lead us not to further replicate this experiment.

We next examined if the effects of BDPP treatment could be explained by a subset of neuroactive metabolites derived from the mixture. Two metabolites from the BDPP cocktail, dihydrocaffeic acid (DHCA) and malvidin-3′-*O*-glucoside (Mal-gluc) were previously reported as key mediators of the behavioral and epigenetic effects of BDPP^16^. Thus mice were treated either with control water or Mal-gluc & DHCA treated water for two weeks prior to 5 and 15 mg/kg morphine CPP (Fig. 6C). For these experiments there was no main effect of metabolite treatment (Fig. 6D—F_(1,27)_ = 2.1; *p* = 0.16), a trend towards main effect of dose (F_(1,27)_ = 3.33; *p* = 0.08), and no significant dose x treatment interactions (F_(1,27)_ = 0.81; *p* = 0.38). These data demonstrate that the effects of the BDPP on addiction-like behaviours are not easily explained by a specific subset of BDPP derived metabolites and may require a more complex mixture of metabolites.

## Discussion

Here we demonstrate treatment with a bioavailable oral polyphenol mixture (BDPP) has marked effects on both the molecular and behavioral responses to morphine. Pretreatment with polyphenols reduces both acute and sensitized locomotor response to high dose morphine, with minimal effects on low dose sensitization (Fig. 1). On morphine CPP, BDPP pretreatment markedly reduced formation of preference at low dose morphine, but potentiated it at high doses (Fig. 2). When we examined the NAc, we found transcriptional effects of morphine were potentiated by BDPP treatment particularly at the high dose of morphine (Fig. 3). Detailed examination of gut microbiome composition and function reinforced significant morphine dose by polyphenol interactions, with robust effects of polyphenol treatment seen after low but not high dose morphine (Fig. 4). Moreover, correlation analysis identified significant negative correlations between CPP score and phyla Bacteroidetes and Proteobacteria, as well as CPP score and genus *Muribibaculaceae* and *Parasutterella* (Fig. 5). Multiple mechanistic studies consequently identified that these myriad effects were not simply explained by SIRT1 activation or by a subset of BDPP derived metabolites (Fig. 6). Taken together, we find polyphenol treatment has both marked and complex effects on the behavioral and physiological response to morphine. These studies lay foundations for future translational work examining the role of dietary phytochemicals in opioid use disorders.

Based on review of the literature, these studies are the first to examine the effects of polyphenols on reward related behaviors in an opioid model. Multiple studies demonstrate that polyphenols and particularly resveratrol can reduce the development of tolerance for morphine, and have anti-nociceptive effects in models of pain^26–28,60–63^. Additionally, oral consumption of BDPP was previously reported to attenuate pain associated with lumbar interverterbral disc injury in rats^24^. While our experiments did not address issues of tolerance or analgesia from morphine, it is possible that the underlying mechanisms are similar, and future work in this area can assess overlap in these areas. Importantly, our model does not result in any physical dependence, tolerance, or withdrawal symptoms at any measurable level, as these phenomenon generally require higher and/or escalating doses over longer periods of time.

Several studies have examined the effects of polyphenols on models of stimulant use disorders with conflicting results. A study examining the effects of SIRT1 on behavioral responses to cocaine found resveratrol enhances cocaine CPP via a SIRT1 dependent mechanism^64^. However, this effect was tested only at a single (5 mg/kg) dose of cocaine, and the resveratrol treatment differed markedly from the one used in our studies. Conversley, resveratrol was also reported to reduce the formation of CPP for high dose cocaine (15 mg/kg)^65^. These seemingly opposing effects on cocaine related behaviors lend further evidence to complex drug dose x polyphenol effects on addiction-like behaviours as observed in the current study (Figs. 1, 2). Such findings indicate the effects of polyphenols on addiction-related behaviours are likley influenced by factors including the drug of abuse in question, dose, chronic or acute exposure, polyphenol used and route of administration.

There is a wealth of literature demonstrating that prolonged changes in gene expression in key limbic brain structures underlie behavioral and synaptic plasticity in models of opioid and other substance use disorders^7,8^. Here, we find that treatment with the BDPP alters gene expression patterns in the NAc, paricularly in response to high dose morphine (Fig. 3). It is important to note with these findings that gene expression changes were measured in response to morphine in a conditioned context, which has been shown to be an important distinction from home cage morphine in previously published work. The exact mechanism of the gene expression changes we see following BDPP and high dose morphine are not clear. Previous studies have reported gene expression changes in the brain following polyphenol treatment, focusing on potential epigenetic effects of polyphenols^66^, including regulation of DNMTs and histone deacetylases^66–68^.

In addition to these behavioral and transcriptomic effects, we found effects of BDPP treatment on the contents and function of the gut microbiome—most notably at low dose morphine (Fig. 4). It is established that dietary polyphenols carry prebiotic properties, enhancing the growth of specific bacterial species which elicit health benefits to the host^56,57^. Indeed, we noted increased abundance of beneficial bacteria including *Akkermansia,* as a result of BDPP treatment in low dose morphine groups (Fig. 4H). Additionally, relative abundance of multiple bacterial phyla and genuses altered by BDPP by morphine dose treatment, correlated with the conditioned place preference response to morphine (Fig. 5). Previous literature has suggested that prebiotic effects of polyphenols may quickly return to baseline after treatment, so it is unclear if our noted effects would last after withdrawal. BDPP induced alterations to the microbiome represent a potential mechanism for the BDPP behavioral responses we report.

While this manuscript characterizes a broad range of effects of BDPP on behavioral and molecular responses for morphine, it is also important to clarify what it does not define. While CPP, and to a lesser extent locomotor sensitization, are utilized as models for substance use disorders the gold standard for this work is voluntary self-administration in animals. Future studies will seek to utilize self-administration to test dose–response, progressive ratio, and drug seeking after abstinence. Additionally, while we find that BDPP has marked effects on the transcriptional profile of the nucleus accumbens in response to morphine in a dose dependent manner (Fig. 3), we find that it does not result in opposite gene regulation across doses, it potentiates the response in the same direction (Fig. S3). Future studies will seek to identify key “driver” genes that may be responsible for behavioral effects, but based on current data we would consider it unlikely that any 1–2 genes are fully responsible for these diverse behavioral and transcriptional effects. Similarly, while the effects of BDPP treatment on microbiome composition are very interesting and suggest potential mechanism (Figs. 4 and 5), it will require significant additional work utilizing gnotobiotic mice to fully plumb the depths of the significance of these findings.

In summary, we demonstrate treatment with dietary polyphenols markedly alters behavioral response to opioids with strong dose x treatment interactions. Dose and treatment effects were also seen on transcriptomic analyiss of the NAc and microbiome sequencing. Mechanistic studies were unable to simplify these effects to SIRT1 activation or a subset of BDPP metabolites. However, importantly, our data demonstrate the capability of dietry polyphenols to modulate behavioural and molecular responses to morphine in a model of opioid use disorder. There remains an urgent need to develop new interventions for treatment of OUD, and in particular non-traditional targets lacking intrinsic abuse potential. The data presented herein lay a strong foundation for the importance of dietary polyphenols as a potential translational research target in OUD, and future studies aim to dissect mechanism, dose, and timing interactions to best harness the beneficial properties of dietary phytochemicals.

## Supplementary Information

Supplementary Tables.

Supplementary Information.
