# Supplementary material for: Dietary polyphenols drive dose-dependent behavioral and molecular alterations to repeated morphine
Source: Sci Rep. 2023 Jul 27;13:12223. doi: 10.1038/s41598-023-39334-9 (PMC10374644; doi:10.1038/s41598-023-39334-9)
Supplement: Supplementary file 2 — Supplementary Information. [file 41598_2023_39334_MOESM2_ESM.pdf]

Supplementary Information

Supplementary Methods

Selection and Presentation of Statistical Cut-offs

The following was a comment in response to reviewer comments during peer review, but we feel it adds important context to our selection of statistical criteria: Importantly, all *p* value cut-offs to determine statistical significance, whether FDR corrected or not, are arbitrary. So, when determining a cut-off for manuscripts we strive to find one that reduces type I error (i.e. no correction) while also not artificially increasing type II error (i.e. over-correction). While many in the field seem to tend towards guarding more stringently against type I error (interpreted as inflating the number of positive findings), scientifically both type I and type II error are equally problematic. In our hands, we have found that the cut-off of an FDR corrected value <0.2 is a good balance between these two issues. Given that this is something we use frequently, a detailed survey of the literature shows a very wide range of FDR correction in published papers ranging from uncorrected $p < 0.05$ as a significance cut-off down to FDR corrected $p < 0.01$. Our hope is that the choice of FDR < 0.2 provides a good balance to reduce either type of error. For Fig. 3K we wanted to highlight changes due to polyphenols between the two morphine groups. Since morphine would be expected to be the strongest driver of transcription between these groups these results are highlighting the smaller changes that occur with polyphenol treatment, so we shifted to an uncorrected $p < 0.01$ to highlight some of these changes. Importantly, this is described very clearly in the text, and full raw and corrected *p* values are provided in the supplemental tables for transparency and reproducibility reasons.

Supplementary Results

Acute and Sensitized Locomotor Responses

Given the reduced response of the BDPP treated group to acute 15mg/kg morphine, we analyzed this response with increased granularity across this session. When the locomotor response is examined in five minute bins across the session, the main effect of treatment immediately becomes clear between the two morphine groups ($F_{(1,10)}=9.98$; $p=0.01$), with post-hoc testing showing that BDPP-treated mice had decreased locomotor activity compared to controls starting in the second bin (**Fig. S1A**). There was no main effect of time across the session ($F_{(8,80)}=1.28$; $p=0.26$) or time x treatment interaction ($F_{(8,80)}=1.11$; $p=0.37$) for the two morphine groups – the saline groups are provided for visual comparison in this panel but not directly compared to morphine groups.

As locomotor sensitization has been shown to persist for weeks, we next performed an experiment in which previously injected mice were given a single challenge injection of morphine two weeks after the conclusion of the initial sensitization experiment. In this context we find that there is no main effect of BDPP treatment (**Fig. S1B** - $F_{(1,35)}=0.07$; $p=0.80$) or significant treatment x dose interaction ($F_{(2,35)}=0.08$; $p=0.92$). As expected, there was a robust effect of morphine dose ($F_{(2,35)}=21.77$; $p<0.0001$). Taken together, these

results suggest that dietary polyphenols have marked effects on locomotor activation in response to morphine, with notable dose and timing interactions.

Conditioned place preference following withdrawal

Since previous studies have demonstrated that conditioned place preference can be potentiated in animals that have previously been treated with morphine [10], we performed CPP after morphine withdrawal as described in **Fig. 2**. However, to ensure that we saw a main effect of morphine withdrawal, we compared effects of CPP completed in previously naïve mice, to those who underwent morphine pretreatment and withdrawal. Here we find that there is indeed a main effect of withdrawal (**Fig. S2** – $F_{(1,69)}=4.48$; $p = 0.03$), as well as BDPP treatment ($F_{(1,69)}=12.57$; $p = 0.0007$), with no significant interactions.

Predicted Functional Enzymatic Pathways

Given the distinct effects of BDPP treatment on microbiome composition in the low and high dose chronic morphine groups, analysis of functional enzymatic pathways differentially affected by BDPP treatment and morphine dose was conducted. Direct comparison of mean sequence proportions between BDPP-5mg/kg and control 5mg/kg morphine groups revealed 222 significantly different pathways (two-tailed Student's t-test and FDR corrected p <0.01). However, direct comparison of mean sequence proportions between BDPP-15mg/kg and control 15mg/kg morphine groups revealed no significantly different pathways using the same cut off thresholds (Full PICRUSt2 list is supplied in **Table S12).** In order to identify which cellular functions the significant enzymes in the 5mg/kg morphine group mapped to, KEGG database was utilized to annotate the significant pathways identified. Interestingly a significant decrease in the mean proportion of enzymes involved in Glycolysis/gluconeogenesis were observed in BDPP 5mg/kg morphine treated mice **(Fig. S4C)**. Meanwhile a significant increase in a number of enzymes involved in Fatty Acid Metabolism and Amino Acid Metabolism was also observed in BDPP 5mg/kg morphine treated mice.

Supplemental Discussion

In addition to models of substance use disorders, polyphenols have also been shown to have marked protective effects in multiple models of neuropsychiatric disorders[11,12]. For example, in models of Alzhimers disease (AD) polyphenolic compounds from a variety of diverse sources have the ability to improve cognitive function and reduce neuropathology in animal models of AD through multiple mechanismsm including improving synaptic plasticity [13]. In particular, a combination of resveratrol, GSPE and concord grape juice have been shown to synergistically mitigate Amyloid β peptide -mediated neuropathology and cognitive impairments in the brain in a mouse model of AD [14]. In addition to effects on neurodegenerative diseases, there have also been a number of studies showing effects of polyphenols

on models of depression. Specifically, chronic treatment with BDPP was found to protect against stress induced depression in mice via an upregulation of VGF in the hippocampus [15], similar to more traditional antidepressants[16]. Chronic BDPP treatment was also reported to alleviate chronic social defeat stress-induced social avoidance via two BDPP-derived metabolites, Mal-Gluc and DHCA, which were identified to promote resilience by modulating synaptic plasticity and peripheral inflammation [17]. Taken together, this body of literature describes important effects of phytochemicals in modulating neuroplasticity in a myriad of neuropsychiatric conditions.

Regarding effects on the microbiome and its functional output, dietary polyphenols have been shown to promote generation of short chain fatty acids (SCFAs) [18]. These byproducts of bacterial fermentation of fibers have numerous effects on brain and behavior [19]. Importantly, previous work from our lab has shown that the presence of SCFA's is critical for behavioral responses to both cocaine and opioids [20,21]. It is possible that behavioral effects of BDPP treatment could be caused by polyphenols' production of key microbial metabolites such as the SCFAs. Indeed, predictive functional analysis of 16s data revealed a significant increase in enzymes involved in SCFA metabolism in the low dose morphine BDPP treated group compared to control counter parts (Fig.**S4C**). Additionally, polyphenols are known to possess antibacterial effects and have been shown to decrease the Firmicutes/Bacteroidetes ratio [22]. This is in line with the results from the current study showing a decrease in Firmicutes/Bacteroidetes ratio following BDPP treatment at low dose morphine (**Fig. 4G**).

Supplementary References

1. Torre D, Lachmann A, Ma'ayan A. BioJupies: Automated Generation of Interactive Notebooks for RNA-Seq Data Analysis in the Cloud. Cell Syst. 2018;7:556-561.e3.
2. Xia J, Gill EE, Hancock REW. NetworkAnalyst for statistical, visual and network-based meta-analysis of gene expression data. Nat Protoc. 2015;10:823–844.
3. Zhou G, Soufan O, Ewald J, Hancock REW, Basu N, Xia J. NetworkAnalyst 3.0: a visual analytics platform for comprehensive gene expression profiling and meta-analysis. Nucleic Acids Res. 2019;47:W234–W241.
4. Raudvere U, Kolberg L, Kuzmin I, Arak T, Adler P, Peterson H, et al. g:Profiler: a web server for functional enrichment analysis and conversions of gene lists (2019 update). Nucleic Acids Res. 2019;47:W191–W198.
5. Chen EY, Tan CM, Kou Y, Duan Q, Wang Z, Meirelles GV, et al. Enrichr: interactive and collaborative HTML5 gene list enrichment analysis tool. BMC Bioinformatics. 2013;14:128.
6. Kuleshov MV, Jones MR, Rouillard AD, Fernandez NF, Duan Q, Wang Z, et al. Enrichr: a comprehensive gene set enrichment analysis web server 2016 update. Nucleic Acids Res. 2016;44:W90–W97.
7. Magoč T, Salzberg SL. FLASH: fast length adjustment of short reads to improve genome assemblies. Bioinformatics. 2011;27:2957–2963.
8. Caporaso JG, Kuczynski J, Stombaugh J, Bittinger K, Bushman FD, Costello EK, et al. QIIME allows analysis of high-throughput community sequencing data. Nat Methods. 2010;7:335–336.
9. Douglas GM, Maffei VJ, Zaneveld JR, Yurgel SN, Brown JR, Taylor CM, et al. PICRUSt2 for prediction of metagenome functions. Nat Biotechnol. 2020;38:685–688.
10. Simpson GR, Riley AL. Morphine preexposure facilitates morphine place preference and attenuates morphine taste aversion. Pharmacol Biochem Behav. 2005;80:471–479.

11. Zhao W, Wang J, Bi W, Ferruzzi M, Yemul S, Freire D, et al. Novel application of brain-targeting polyphenol compounds in sleep deprivation-induced cognitive dysfunction. Neurochem Int. 2015;89:191–197.
12. Ho L, Ferruzzi MG, Janle EM, Wang J, Gong B, Chen T-Y, et al. Identification of brain-targeted bioactive dietary quercetin-3-O-glucuronide as a novel intervention for Alzheimer's disease. FASEB J Off Publ Fed Am Soc Exp Biol. 2013;27:769–781.
13. Wang J, Ferruzzi MG, Ho L, Blount J, Janle EM, Gong B, et al. Brain-targeted proanthocyanidin metabolites for Alzheimer's disease treatment. J Neurosci Off J Soc Neurosci. 2012;32:5144–5150.
14. Wang J, Bi W, Cheng A, Freire D, Vempati P, Zhao W, et al. Targeting multiple pathogenic mechanisms with polyphenols for the treatment of Alzheimer's disease-experimental approach and therapeutic implications. Front Aging Neurosci. 2014;6:42.
15. Jiang C, Sakakibara E, Lin W-J, Wang J, Pasinetti GM, Salton SR. Grape-derived polyphenols produce antidepressant effects via VGF- and BDNF-dependent mechanisms. Ann N Y Acad Sci. 2019;1455:196–205.
16. Jiang C, Lin W-J, Sadahiro M, Labonté B, Menard C, Pfau ML, et al. VGF function in depression and antidepressant efficacy. Mol Psychiatry. 2018;23:1632–1642.
17. Wang J, Hodes GE, Zhang H, Zhang S, Zhao W, Golden SA, et al. Epigenetic modulation of inflammation and synaptic plasticity promotes resilience against stress in mice. Nat Commun. 2018;9:477.
18. Frolinger T, Sims S, Smith C, Wang J, Cheng H, Faith J, et al. The gut microbiota composition affects dietary polyphenols-mediated cognitive resilience in mice by modulating the bioavailability of phenolic acids. Sci Rep. 2019;9:3546.
19. Dalile B, Van Oudenhove L, Vervliet B, Verbeke K. The role of short-chain fatty acids in microbiota-gut-brain communication. Nat Rev Gastroenterol Hepatol. 2019;16:461–478.
20. Hofford RS, Mervosh NL, Euston TJ, Meckel KR, Orr AT, Kiraly DD. Alterations in microbiome composition and metabolic byproducts drive behavioral and transcriptional responses to morphine. Neuropsychopharmacol Off Publ Am Coll Neuropsychopharmacol. 2021. 14 June 2021. https://doi.org/10.1038/s41386-021-01043-0.
21. Kiraly DD, Walker DM, Calipari ES, Labonte B, Issler O, Pena CJ, et al. Alterations of the Host Microbiome Affect Behavioral Responses to Cocaine. Sci Rep. 2016;6:35455.
22. Barbieri R, Coppo E, Marchese A, Daglia M, Sobarzo-Sánchez E, Nabavi SF, et al. Phytochemicals for human disease: An update on plant-derived compounds antibacterial activity. Microbiol Res. 2017;196:44–68.

Supplementary Figures

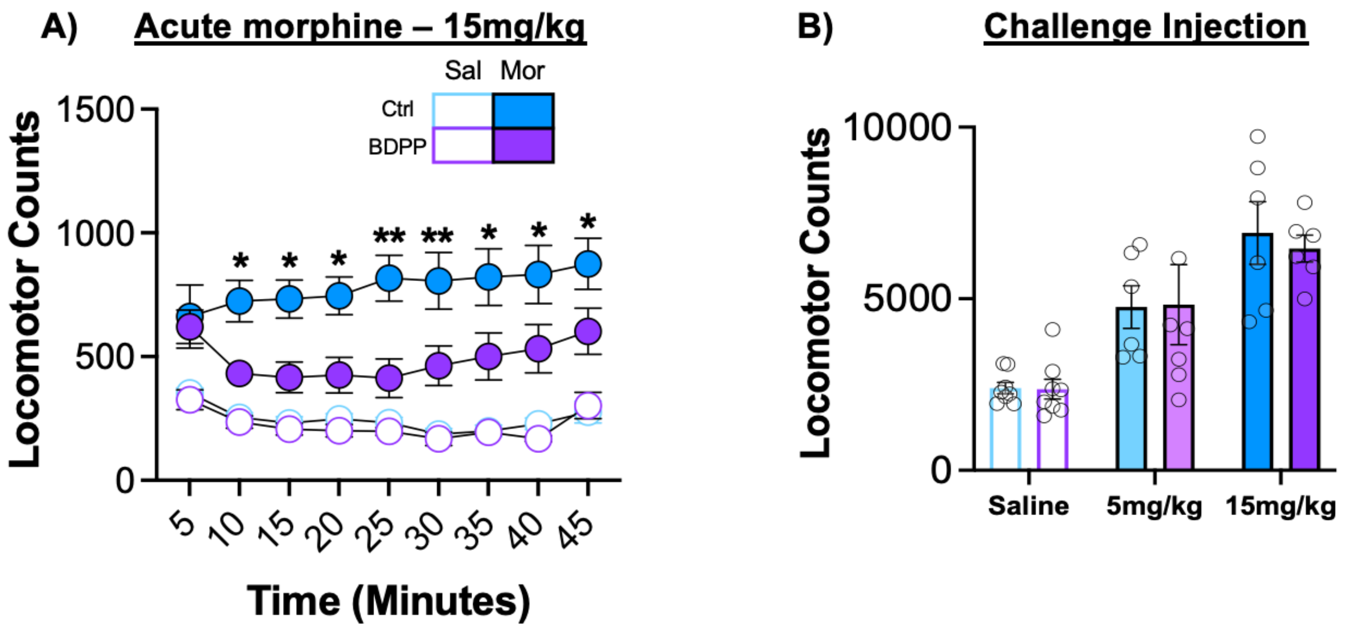

Figure S1 – Acute and Sensitized Locomotor Responses. **(A)** Acute effects of morphine on locomotor activation were also decreased by pre-treatment with BDPP polyphenols. **(B)** In mice previously treated with 5 days of morphine, polyphenol treatment did not affect persistence of morphine sensitization after two weeks of withdrawal.

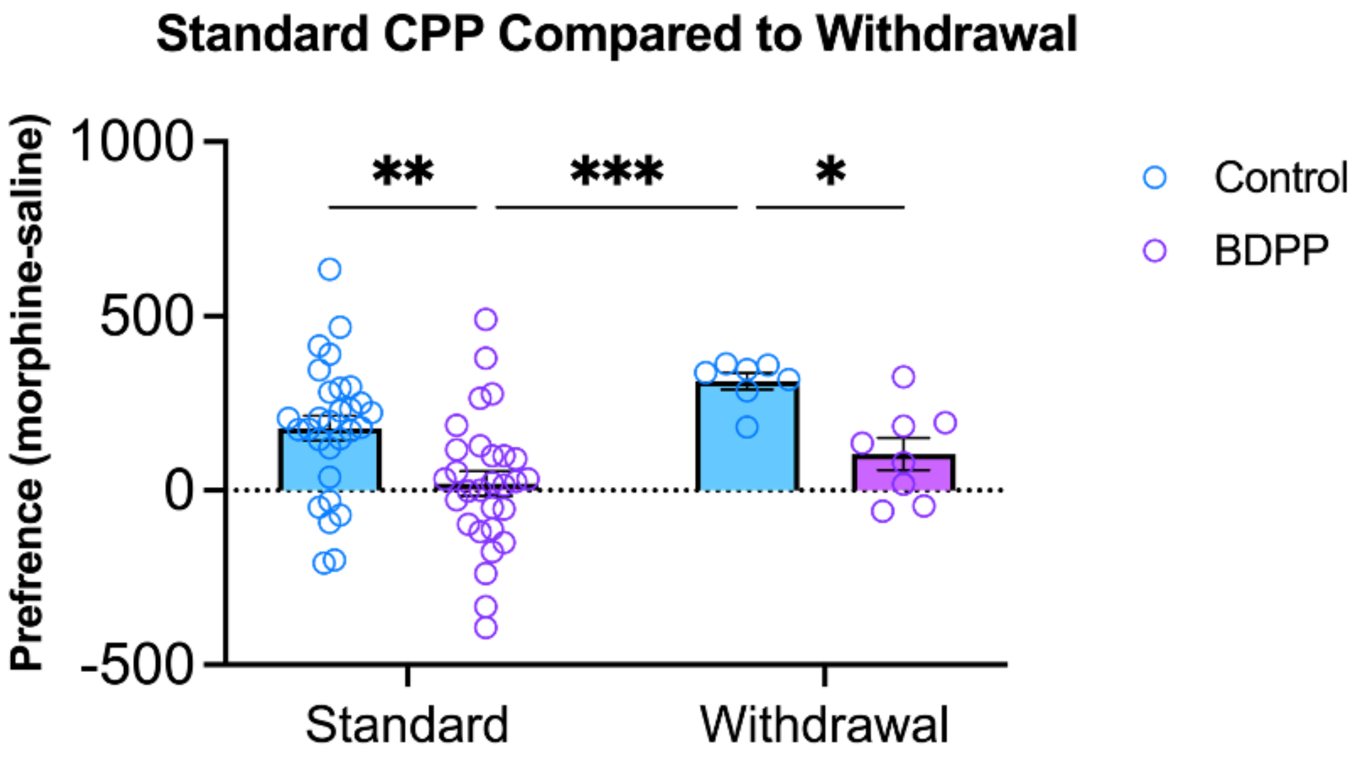

ANOVA table	SS (Type III)	DF	MS	F (DFn, DFd)	P value
Interaction	7473	1	7473	F (1, 69) = 0.2340	P=0.6301
* Withdrawal	142914	1	142914	F (1, 69) = 4.475	P=0.0380
** Treatment	401448	1	401448	F (1, 69) = 12.57	P=0.0007

Figure S2 – Effect of morphine pretreatment on subsequent development of conditioned place preference. Mice that were pretreated with morphine followed by two weeks of withdrawal prior to CPP conditioning demonstrated more robust acquisition of conditioned place preference on subsequent training.

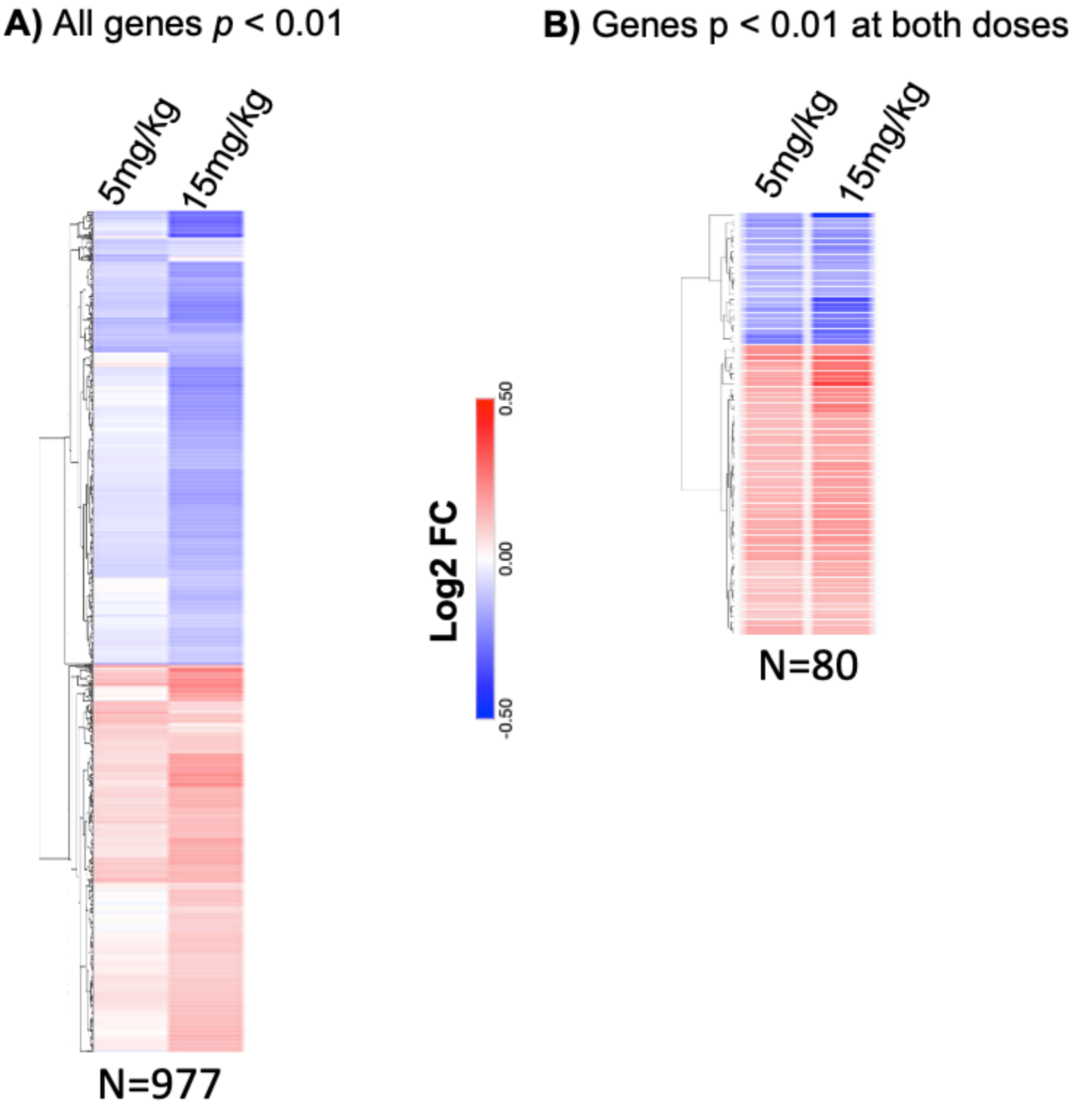

Figure S3 – Analysis of genes with *p < 0.01* in BDPP groups compared to saline. Given the differential effects of BDPP treatment on behavior at the two different morphine doses, we examined patterns of gene expression in BDPP treated animals compared to saline controls at both doses. (A) Heatmap of all genes with a *p* value < 0.01 in either BDPP group relative to control saline animals. While no genes were regulated in opposite directions, the effects on gene expression are more robust at 15mg/kg. (B) Heatmap of all genes with a *p* value < 0.01 in both the 5mg/kg and 15mg/kg morphine treatment groups relative to control saline. Here, the same pattern emerges with more robust effects on expression at 15mg/kg, but no overtly discrepant changes in expression.

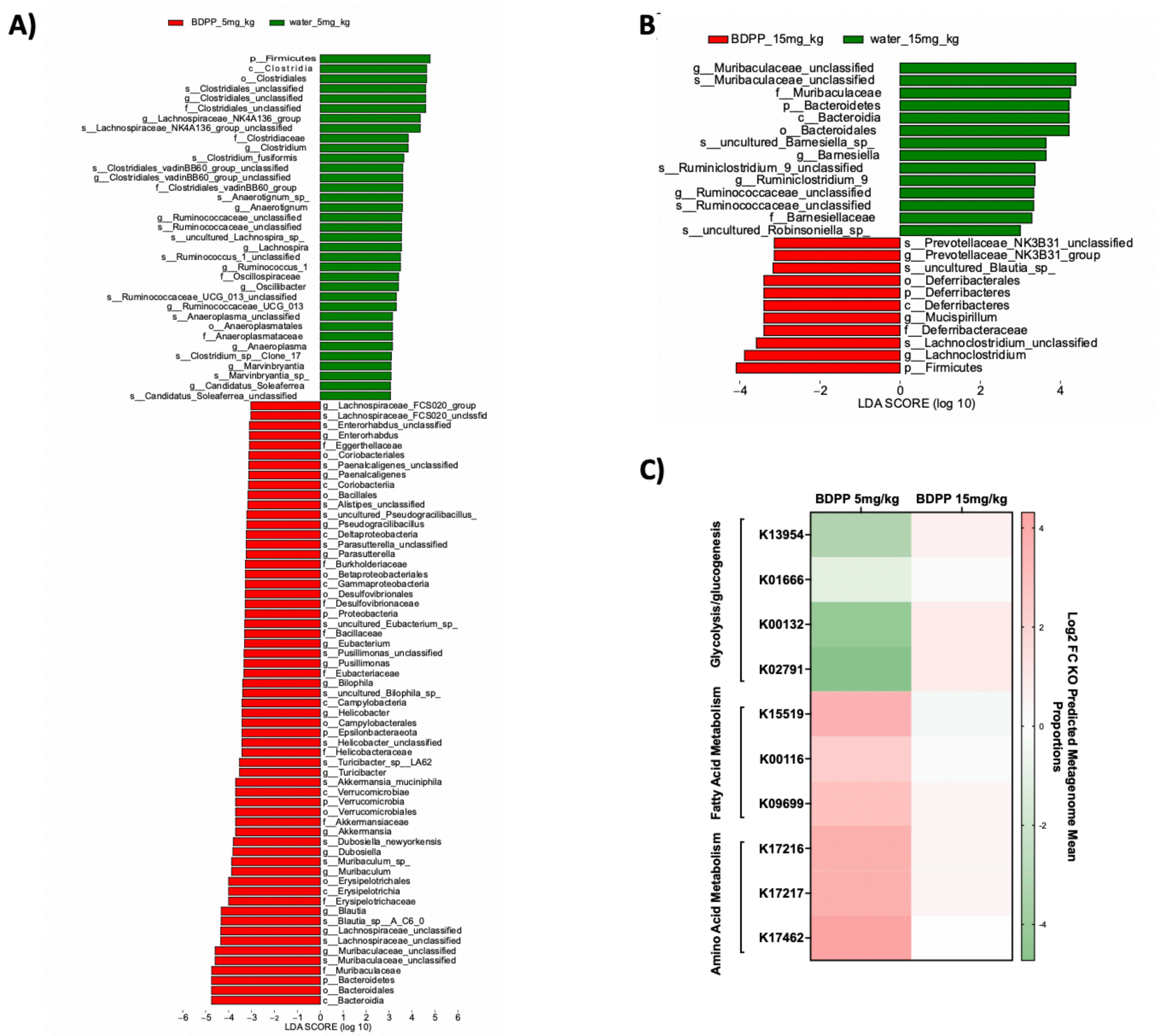

Figure S4– BDPP Treatment Differentially Regulates Microbiome Composition and Functional Output in Low and High Dose Morphine Treated Animals. Linear discriminant analysis (LDA) effect size (LEfSe) analysis of 16S data was used to determine bacterial taxa which are differentially represented between treatment groups **(A)** list of bacterial taxa found to be overrepresented (Red) or underrepresented (Green) in BDPP 5gm/kg relative to control 5mg/kg treatment groups **(B)** list of bacterial taxa found to be overrepresented (Red) or underrepresented (Green) in BDPP 15mg/kg relative to control 15mg/kg treatment groups **(C)** Phylogenetic Investigation of Communities by Reconstruction of Unobserved States (PICRUSt2) package identified enzymes predicted to be involved in glycolysis/glucogenesis to be significantly decreased, fatty acid metabolism and amino acid metabolism enzymes increased in BDPP 5mg/kg treated animals relative to controls (FDR corrected *p* <0.01 significance cut off).These same enzymatic pathways were unchanged in BDPP 15mg/kg treated animals.
